# Editorial: Cannabinoids in neuroinflammation, neurodegeneration and pain: Focus on non-neuronal cells

**DOI:** 10.3389/fnins.2022.1114775

**Published:** 2022-12-20

**Authors:** Haley A. Vecchiarelli, Valerie Joers, Malú Gámez Tansey, Katarzyna Starowicz

**Affiliations:** ^1^Division of Medical Sciences, University of Victoria, Victoria, BC, Canada; ^2^Department of Neuroscience, Center for Translational Research in Neurodegenerative Disease, University of Florida, Gainesville, FL, United States; ^3^Department of Neurology, Norman Fixel Institute for Neurological Diseases, University of Florida Health, Gainesville, FL, United States; ^4^Department of Neurochemistry, Maj Institute of Pharmacology PAS, Kraków, Poland

**Keywords:** cannabinoids, neuroinflammation, neurodegeneration, non-neuronal cells, editorial

Cannabinoids have the potential to be therapeutic for neurological and psychiatric diseases associated with neuroinflammation, neurodegeneration and pain (Kelly et al., 2020; Bryk and Starowicz, 2021). The cannabinoid system includes endogenous cannabinoids, phytocannabinoids and synthetic cannabinoids, their target receptors and biosynthetic and degradative enzymes (Morena et al., 2016). Alterations of the cannabinoid system are associated with the inflammatory processes of these conditions, but intriguingly, are also implicated in the alterations in affect which may co-occur (Vecchiarelli et al., 2021). Non-neuronal cells in the central nervous system are in particular related to the pathogenesis, maintenance and/or alleviation of neuroinflammatory, neurodegenerative and pain states (Kelly et al., 2020; Šimončičová et al., 2022; St-Pierre et al., 2022). To further elucidate the role of cannabinoids in these disease contexts, this Research Topic includes a collection of primary research articles and a mini review on the role of cannabinoids in these states in particular in non-neuronal cells.

Two primary research articles in the collection investigate the role of enzymes that metabolize *N*-acylethanolamines, including the primary endocannabinoid, *N*-arachidonoylethanolamine (anandamide or AEA). In Duncan et al., human neural precursor cell culture (ReN cells) were exposed to sublethal oxidative stress [tert-butyl hydroperoxide (tBHP)]—which is particularly associated with neurodegenerative diseases. They found that exposure to tBHP increased protein levels of cannabinoid receptors (CB1 and CB2) and of an *N*-acylethanolamine metabolizing enzyme, fatty acid amide hydrolase (FAAH), which metabolizes AEA, as well as the associated *N*-acylethanolamine molecules, oleoylethanolamide (OEA) and palmitoylethanolamide (PEA) (Malek and Starowicz, 2016). However, exposure to a lower dose of tBHP increased expression of *N*-acylethanolamide specific phospholipase D (NAPE-PLD), a synthesizing enzyme for AEA. Intriguingly, they also found that the mild level of oxidative stress also increased neurite outgrowth. It is possible that because AEA can serve to promote neurite outgrowth (Compagnucci et al., 2013), that the oxidative stress-induced increases in NAPE-PLD generated AEA, leading to the observed increased neurite outgrowth. It is possible these cannabinoid increases are protective, as previously demonstrated (Elmazoglu et al., 2020). While Duncan et al., showed an effect of oxidative stress on the *N*-acylethanolamine metabolizing enzyme, FAAH, Vecchiarelli et al., investigates the effects of a single nucleotide polymorphism (SNP) in *FAAH* (C385A) mouse model (Dincheva et al., 2015) on basal and colitis-induced alterations in inflammatory mediators in plasma and the amygdala. Carriers of the mutant allele have reduced FAAH activity and show attenuated colitis-induced increases of plasma IL-2, LIF, MCP-1, and TNF, as well as amygdala G-CSF and MCP-1 levels—without altering the colitis-induced disease macroscopic colon damage. Interestingly, following chronic stress, the receptor for MCP-1 is necessary for the development of anxiety-like behavior-inducing monocyte trafficking to the brain (Wohleb et al., 2013). Additionally, a central increase of FAAH activity and decrease of AEA levels can contribute to colitis-induced anxiety (Vecchiarelli et al., 2021); therefore, it is possible that colitis-induced reductions of AEA allow for an increase in MCP-1 contributing to monocyte-trafficking-induced generation of anxiety-like behavior, suggesting AEA is a potential modulator of inflammatory responses. Additionally, Vecchiarelli et al., show that FAAH reduction leads to reduced IL-1α, IL-9, MIP-1β, and MIP-2 levels in the amygdala, indicating that FAAH, or the compounds it metabolizes, may be involved in their baseline regulation centrally. Together, these studies illustrate the effects of FAAH in response to oxidative stress and on neuroinflammation, which may be important for neurodegeneration and the affective symptoms of inflammatory diseases.

The remaining articles in this Research Topic discuss the role of CB2. Honig et al., show the effects of a clinically available CB2 inverse agonist, Raloxifene, on visual system outcomes following focal cranial impact mild traumatic brain injury (mTBI). They found that Raloxifene reversed the effects of mTBI on contrast sensitivity, light aversion, pupillary excessive dilation and optic nerve axonal loss. Furthermore, in the injured optic nerve, Honig et al., show that IBA1+ cell numbers are increased and have a normalized transcriptional profile after 10 mg/kg of Raloxifene following mTBI. Therefore, highlighting a further protective role for CB2 inverse agonism in the regulation of IBA1+ cells (predominantly microglia) following mTBI, which may contribute to the beneficial outcomes seen in visual behavior following mTBI. The protective effects of inverse agonism might seem counterintuitive, as inverse agonists suppress constitutive activity and activation of CB2 has shown a role in cytokine signaling and is generally considered anti-inflammatory (Young and Denovan-Wright, 2021), although this is not always the case, as there may be ligand-specific signaling biases (Oláh et al., 2017). Important to the function of microglia and myeloid cells, activation of CB2 can suppress phagocytosis (Han et al., 2022). Therefore, it is possible that CB2 inverse agonism promotes favorable disease outcomes by accelerating phagocytosis allowing the innate immune system to clear debris while also providing an environment enriched in restorative mediators (Yu et al., 2020; Martinez Ramirez et al., 2022). This indicates that characterization of Raloxifene and other CB2 inverse agonists at microglial CB2 could be of great benefit for therapeutic discovery.

Our final article is a mini-review by Ferranti and Foster, which highlights a role for CB2 in schizophrenia, a disease becoming increasingly understood to possess inflammatory risk factors (Comer et al., 2020a) and microglia-mediated mechanisms (Sekar et al., 2016; Comer et al., 2020b). This article highlights that in addition to, or maybe through, inflammatory mediator signaling, a role for CB2 receptors in microglia on associated behaviors, such as contextual fear memory. Fear memory was enhanced with the overexpression of CB2 in hippocampal CA1 microglia, yet reduced with disrupted microglial CB2 (Li and Kim, 2017). The Ferranti and Foster review reminds us that CB2 receptors have also been described on some neuronal populations. Therefore, a crucial next step in the field is to better understand the cellular distribution of central CB2 and the interplay between neuronal and non-neuronal CB2 under basal physiological conditions and across disease phenotypes.

Further understanding of the complex signaling of this system will hopefully lead to the generation or refinement of therapeutics for a host of neurological and psychiatric diseases and articles in this collection have contributed toward this goal.

## Author contributions

HV and VJ wrote the initial draft of the manuscript. All authors contributed to manuscript revision, read, and approved the submitted version.
